# Geniposide Mitigates Insulin Resistance and Hepatic Fibrosis via Insulin Signaling Pathway

**DOI:** 10.3390/ijms26168079

**Published:** 2025-08-21

**Authors:** Seung-Hyun Oh, Min-Seong Lee, Byung-Cheol Lee

**Affiliations:** Department of Clinical Korean Medicine, Graduate School, Kyung Hee University, 26 Kyungheedae-ro, Dongdaemun-gu, Seoul 02447, Republic of Korea; sshyunbiz@naver.com

**Keywords:** geniposide, insulin resistance, non-alcoholic steatohepatitis, insulin signaling pathway

## Abstract

Insulin resistance is a key driver of metabolic disorders, including type 2 diabetes and non-alcoholic fatty liver disease (NAFLD), progressing to non-alcoholic steatohepatitis (NASH). This study investigated the effects of geniposide (GP) on insulin sensitivity and hepatic fibrosis in a high-fat diet (HFD)-induced NASH model. C57BL/6 mice were fed an HFD for five weeks and subsequently divided into normal chow (NC), HFD, HFD with GP 50 mg/kg (GP50), and HFD with GP 100 mg/kg (GP100) groups. The treatments were administered orally for 12 weeks. GP treatment significantly reduced body weight as well as epididymal fat and liver weights, while no differences were observed in food intake. Improvements in glucose and lipid metabolism were observed in oral glucose tolerance tests, homeostatic model assessment of insulin resistance (HOMA-IR), and blood lipid profiles. Histological analyses revealed that GP suppressed adipocyte hypertrophy and hepatic lipid accumulation and hepatic fibrosis. To further elucidate molecular mechanisms of GP, quantitative real-time polymerase chain reaction (qRT-PCR) analysis was conducted in the liver tissue. GP downregulated expression of inflammatory markers, including F4/80, tumor necrosis factor (TNF)-α, and interleukin (IL)-6. GP treatment modulated genes involved in insulin signaling including Janus kinase 2 (JAK2), insulin receptor (INSR), insulin receptor substrate 2 (IRS-2), and protein kinase B (AKT1) gene expression levels. This suggests GP suppresses inflammation and mitigates insulin resistance by activating the INSR–IRS2–Akt pathway. Additionally, GP enhanced adenosine monophosphate-activated protein kinase (AMPK) expression, suggesting its potential role in improving glucose and lipid metabolism. In conclusion, GP improves insulin resistance, inflammation, and hepatic fibrosis, highlighting its therapeutic potential for NASH and related metabolic disorders.

## 1. Introduction

Insulin resistance, characterized by the diminished ability of cells to respond to insulin, significantly increases the risk of developing chronic metabolic disorders, including type 2 diabetes mellitus and non-alcoholic fatty liver disease (NAFLD), which can progress to non-alcoholic steatohepatitis (NASH)—a severe liver disease marked by inflammation, fibrosis, and increased risk of cirrhosis and hepatocellular carcinoma—contributing to substantial morbidity and mortality worldwide. Obesity is a major contributor to insulin resistance, inducing chronic low-grade inflammation across various tissues through pro-inflammatory cytokines and adipokines primarily released from adipose tissue [1]. Moreover, inflammation plays a crucial role in the progression of insulin resistance and NASH, creating a vicious cycle in which metabolic dysfunction exacerbates hepatic injury and fibrosis [2]. Despite continued advances in therapeutic development, the global prevalence of diabetes has been steadily rising [3]. Given that insulin resistance is a key pathophysiological feature of both type 2 diabetes and NAFLD/NASH, the sharp increase in diabetes prevalence indicates a parallel rise in global metabolic dysfunction, underscoring the urgent need for the development of novel therapeutic agents against insulin resistance.

Geniposide (GP), an iridoid glycoside primarily extracted from the fruit of *Gardenia jasminoides*, is known for its beneficial effects on metabolic disorders associated with inflammation and insulin resistance. GP has been studied for its ability to regulate immune responses by reducing T helper 17 cell cytokines, such as interleukin (IL)-2, while increasing regulatory T-cell cytokines, including IL-4 and transforming growth factor beta 1 (TGF-β1), in mesenteric lymph node lymphocytes [4]. Wu et al. reported that GP exerts a hypoglycemic effect by modulating hepatic glucose-metabolizing enzymes, such as hepatic glycogen phosphorylase and glucose-6-phosphatase (G6Pase) [5]. Additionally, GP has been shown to ameliorate liver fibrosis by inhibiting oxidative stress, reducing inflammatory responses, and decreasing apoptosis in hepatic cells [6]. Despite these promising findings, few studies have investigated the effects of GP on NASH, and they were primarily focused on hepatic inflammation, peroxisome proliferator-activated receptor α (PPAR-α), and oxidative stress [7]. This is the first study to demonstrate that GP ameliorates NASH by enhancing insulin sensitivity through modulation of the insulin signaling pathway in high-fat diet (HFD)-induced NASH mice model. This study integrates histological and molecular approaches, including liver and adipose tissue histology and mRNA expression profiling, to comprehensively evaluate the therapeutic potential of GP in metabolic dysfunction and NASH.

## 2. Results

### 2.1. Effects of GP on Weight Gain

**Figure 1:**
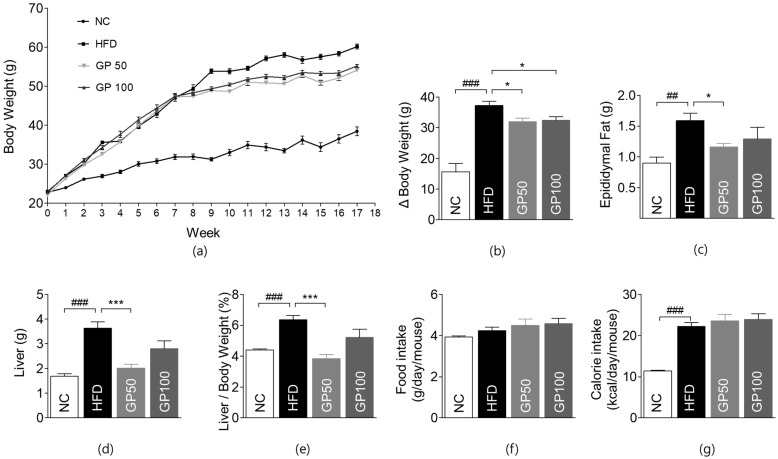
GP reduces body, epididymal fat pad, and liver weights in HFD-fed mice. (**a**) Weekly body weight (g). (**b**) Body weight change (g). (**c**) Weight of epididymal fat pads (g). (**d**) Weight of liver (g). (**e**) Ratio of liver weight to total body weight (%). (**f**) Food intake (g/day/mouse). (**g**) Calorie intake (kcal/day/mouse). Data are described as the mean ± SEM; ## *p* < 0.01, ### *p* < 0.001 compared with NC. * *p* < 0.05, *** *p* < 0.001 compared with HFD. NC, normal chow (*n* = 5); HFD, high-fat diet (*n* = 5); GP50, HFD + geniposide 50 mg/kg/day (*n* = 5); GP100, HFD + geniposide 100 mg/kg/day (*n* = 5).

Treatment with GP has significantly reduced weight gain from baseline than that with HFD. The mean change in body weight from baseline to week 17 was significant in the GP50 and GP100 groups compared to the HFD group (Figure 1a,b). We evaluated the weight change in epididymal fat pads and liver, which are insulin-sensitive organs. At 17 weeks, the GP50 group showed significant suppression in epididymal fat pad weight compared to the HFD group (Figure 1c). Significant suppression of the increase in liver weight and liver weight ratio was observed in the GP50 group as compared with HFD (Figure 1d,e).

The GP group showed no differences in daily food intake and calorie intake from the HFD control group, suggesting that GP’s anti-obesity effect was not linked to food intake (Figure 1f,g).

### 2.2. Effect of GP on Glucose Metabolism

**Figure 2:**
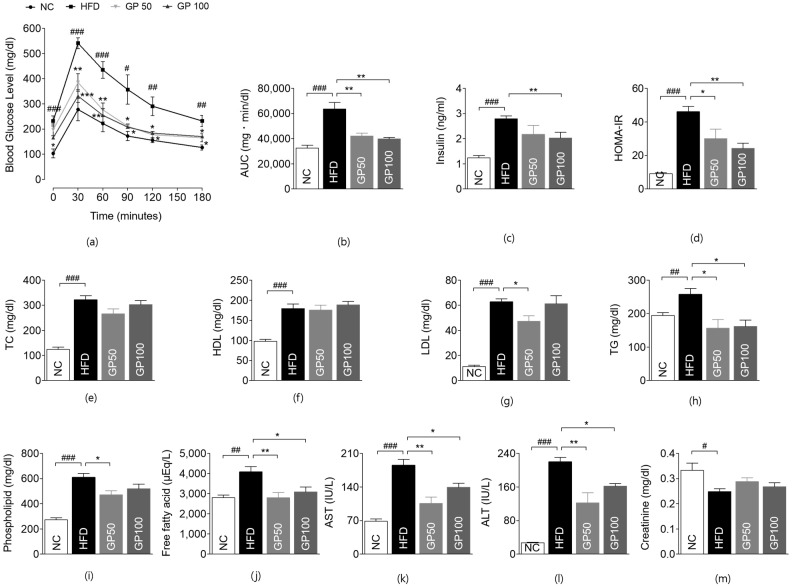
GP ameliorates glucose intolerance, dyslipidemia, and hepatic dysfunction in HFD-fed mice. (**a**) OGTT (oral glucose tolerance test) (mg/dL); (**b**) AUC (area under curve) (mg·min/dL); (**c**) insulin (ng/mL); (**d**) HOMA-IR (homeostatic model assessment of insulin resistance); (**e**) TC (total cholesterol) (mg/dL); (**f**) HDL-C (high-density lipoprotein cholesterol) (mg/dL); (**g**) LDL-C (low-density lipoprotein cholesterol) (mg/dL); (**h**) TG (mg/dL); (**i**) phospholipid (mg/dL); (**j**) free fatty acid (μEq/L); (**k**) AST (aspartate aminotransaminase) level (IU/L); (**l**) ALT (alanine aminotransaminase) level (IU/L); (**m**) creatinine level (mg/dL). Data are described as the mean ± SEM; # *p* < 0.05, ## *p* < 0.01, ### *p* < 0.001 compared with NC, * *p* < 0.05, ** *p* < 0.01 compared with HFD. NC, normal chow (*n* = 5); HFD, high-fat diet (*n* = 5); GP50, HFD + geniposide 50 mg/kg/day (*n* = 5); GP100, HFD + geniposide 100 mg/kg/day (*n* = 5).

To explore the effect of GP on glucose metabolism, an oral glucose tolerance test (OGTT) was conducted at week 17. At each time point, the HFD group presented higher blood glucose levels than the NC group. GP treatment reduced glucose levels overall compared to the HFD group. Blood glucose levels at 0 min were significantly suppressed in the GP100 group compared to the HFD group. All GP-treated groups showed significant decreases in blood glucose levels at 30, 60, 90, 120, and 180 min compared to the control group (Figure 2a). Both GP groups showed a reduced area under the curve (AUC) compared to the HFD group, with substantial between-group differences (Figure 2b). The fasting insulin level and homeostatic model assessment of insulin resistance (HOMA-IR) calculated from fasting insulin and glucose levels were further evaluated at week 17. Insulin levels and HOMA-IR were significantly elevated in the HFD group compared to the NC group. The GP 100 group showed marked suppression in fasting insulin levels compared to the HFD group (Figure 2c), and HOMA-IR were reduced in the GP treatment groups dose-dependently (Figure 2d).

### 2.3. Effect of GP on Blood Lipid Profile

We evaluated the lipid profile at 17 weeks to observe the impact of GP on lipid metabolism. Lipid levels, including of total cholesterol (TC), high-density lipoprotein cholesterol (HDL-C), low-density lipoprotein cholesterol (LDL-C), triglycerides (TG), phospholipid, and free fatty acid (FFA) increased significantly in the HFD group as compared with the NC group (Figure 2e–j). The LDL-C level was significantly lower in the GP50 group compared to the HFD group (Figure 2g). TG and FFA levels were significantly improved in both GP groups compared to the HFD group (Figure 2e,j). The phospholipid level was significantly lower in the GP50 group compared to the HFD group (Figure 2i).

### 2.4. Effect of GP on Hepatic and Renal Function Tests

Aspartate aminotransferase (AST) and alanine aminotransferase (ALT) levels were significantly increased in the HFD group compared to the NC group, while GP50 and GP100 effectively prevented AST and ALT elevation (Figure 2k,l). Mean creatinine levels at week 17 showed no significant difference between the GP50 and GP100 groups compared to the HFD group (Figure 2m).

### 2.5. Effect of GP on Histological Analysis of Liver and Adipose Tissue

**Figure 3:**
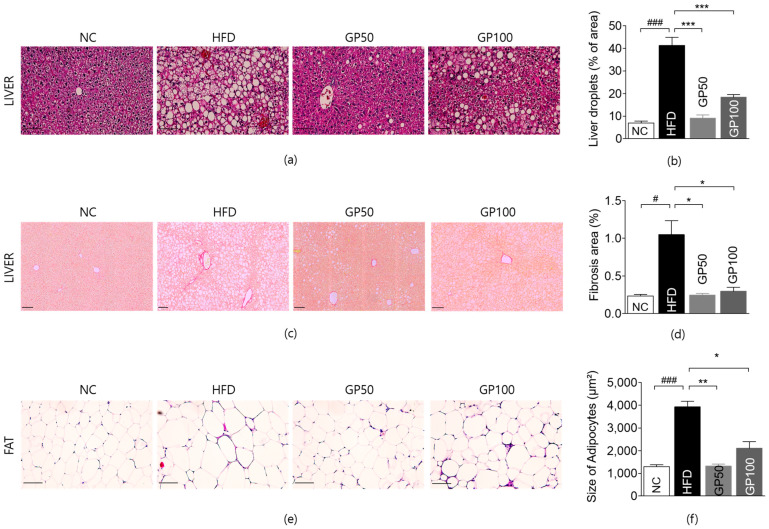
GP ameliorates hepatic lipid accumulation, hepatic fibrosis, and adipocyte hypertrophy in HFD-fed mice. (**a**) H&E staining image of the liver (magnification ×200); (**b**) lipid droplets in the liver (%); (**c**) Sirius red staining image of the liver (magnification ×100); (**d**) fibrosis area in live tissue (%); (**e**) H&E staining image of epididymal fat (magnification ×200); (**f**) size of adipocytes in epididymal fat tissue (µm^2^). Data are described as the mean ± SEM. Bar indicates 100 µm; # *p* < 0.05 compared with NC, ### *p* < 0.001 compared with NC, * *p* < 0.05 compared with HFD, ** *p* < 0.01 compared with HFD, *** *p* < 0.001 compared with HFD. NC, normal chow (*n* = 5); HFD, high-fat diet (*n* = 5); GP50, HFD + geniposide 50 mg/kg/day (*n* = 5); GP100, HFD + geniposide 100 mg/kg/day (*n* = 5).

The size and number of lipid droplets in liver tissue significantly expanded in the HFD group compared to the NC group. The percentage of lipid droplets in liver tissue in the treatment groups showed a significant decrease as compared to the HFD group (Figure 3a,b).

The fibrosis area in the liver tissue of the HFD group was significantly increased compared to the NC group. The liver fibrosis area was significantly reduced in both GP groups compared to the HFD group (Figure 3d,e).

The size of adipocytes in epididymal fat pads in the HFD group was significantly more enlarged than in the NC group. There was substantial suppression in the size of adipocytes in both GP groups compared with the HFD group (Figure 3e,f).

### 2.6. Effect of GP on Gene Expression in Liver Tissue

**Figure 4:**
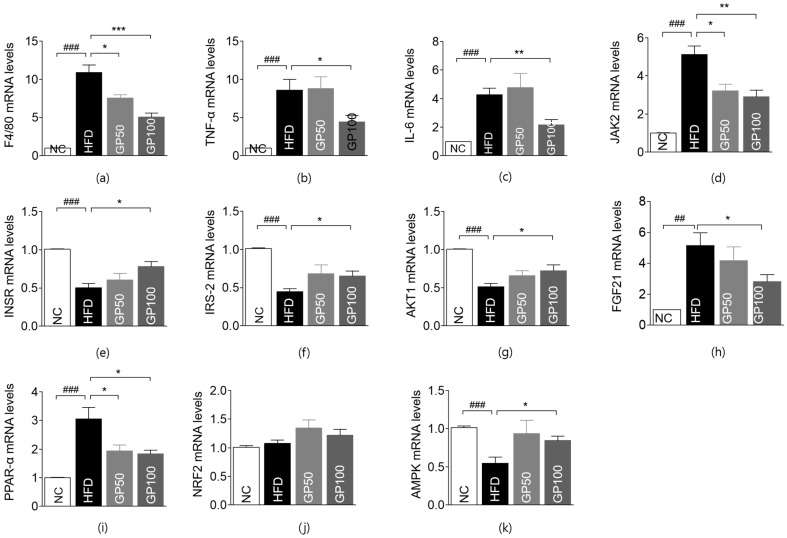
GP modulates hepatic expression of genes related to inflammation, insulin signaling, and metabolism. (**a**) F4/80; (**b**) TNF-α; (**c**) IL-6; (**d**) JAK2; (**e**) INSR; (**f**) IRS-2; (**g**) AKT1; (**h**) FGF21; (**i**) PPAR-α; (**j**) NRF2; (**k**) AMPK. Data are described as the mean ± SEM; ## *p* < 0.01, ### *p* < 0.001 compared with NC, * *p* < 0.05, ** *p* < 0.01, *** *p* < 0.001 compared with HFD. NC, normal chow (*n* = 5); HFD, high-fat diet (*n* = 5); GP50, HFD + geniposide 50 mg/kg/day (*n* = 5); GP100, HFD + geniposide 100 mg/kg/day (*n* = 5); TNF-α, tumor necrosis factor-α; IL-6, Interleukin 6; JAK2, Janus kinase 2; INSR, insulin receptor; IRS-2, insulin receptor substrate 2; AKT1, alpha serine/threonine-protein kinase1; FGF21, fibroblast growth factor 21; PPAR-α, peroxisome proliferator-activated receptor α; NRF2, nuclear factor erythroid 2-related factor 2; AMPK, adenosine monophosphate-activated protein kinase.

The gene expressions of inflammation-related markers, including F4/80, tumor necrosis factor- α (TNF-α), IL-6, IL-10, and interferon-γ (IFN-γ), were significantly upregulated, while the Mitogen-activated protein kinase 14 (MAPK14) level was significantly reduced in the HFD group compared with the NC group (Figure 4a–c, Supplementary Materials). F4/80 levels were significantly attenuated in the GP50 and GP100 groups in a dose-dependent manner compared to the control group (Figure 4a). Moreover, TNF-α and IL-6 expression was significantly suppressed in the GP100 group (Figure 4b,c). The gene expressions of IL-10, IFN-γ, and MAPK14 were insignificant between the treatment and control groups (Figure S1).

Regarding genes related to the insulin signaling pathway, the HFD group exhibited significant alterations in the expression of janus kinase 2 (JAK2), insulin receptor (INSR), insulin receptor substrate 1/2 (IRS1/2), alpha serine/threonine-protein kinase1 (AKT1), and insulin like growth factor 1 receptor (IGF1R) compared to the NC group (Figure 4d–g and Figure S1). Both treatment groups downregulated JAK2 expression in a dose-dependent manner (Figure 4d). GP100 showed a significant increase in the gene expressions of INSR, IRS-2, and AKT1 compared to the HFD group (Figure 4e–g).

The expression of genes related to lipid and glucose metabolism in the liver revealed that fibroblast growth factor 21 (FGF21) and peroxisome proliferator-activated receptor α (PPAR-α) were upregulated, while adenosine monophosphate-activated protein kinase (AMPK) was downregulated in the HFD group relative to the NC group; nuclear factor erythroid 2-related factor 2 (NRF2) expression remained unchanged (Figure 4h–k). GP100 treatment significantly decreased FGF21 expression and increased AMPK expression, while PPAR-α expression was consistently reduced across all GP-treated groups; NRF2 expression remained unchanged among all groups (Figure 4h–k).

### 2.7. Effect of GP on Gene Expression in Adipose Tissue

**Figure 5:**
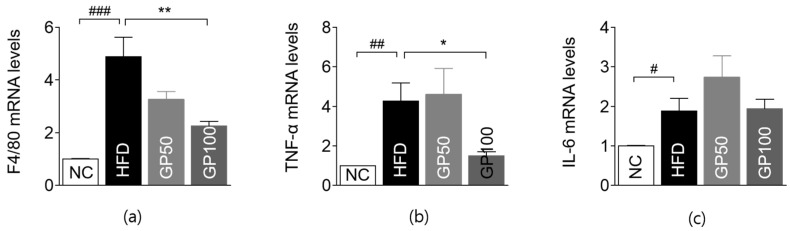
GP attenuates adipose inflammation by modulating inflammatory gene expression. (**a**) F4/80; (**b**) TNF-α; (**c**) IL-6. Data are described as the mean ± SEM; # *p* < 0.05, ## *p* < 0.01, ### *p* < 0.001 compared with NC, * *p* < 0.05, ** *p* < 0.01 compared with HFD. NC, normal chow (*n* = 5); HFD, high-fat diet (*n* = 5); GP50, HFD + geniposide 50 mg/kg/day (*n* = 5); GP100, HFD + geniposide 100 mg/kg/day (*n* = 5); TNF-α, tumor necrosis factor-α; IL-6, Interleukin 6.

F4/80, TNF-α, IL-6, IL-10, and IFN-γ levels were all significantly upregulated in the HFD group compared to the NC group (Figure 5 and Figure S2). In the GP100 group, F4/80 and TNF-α levels were significantly decreased compared to the HFD group (Figure 5a,b).

## 3. Discussion

This study aimed to explore the molecular mechanisms underlying the effects of GP on obesity-related inflammation and insulin resistance and further extended the investigation to NASH. GP treatment enhanced the expression of JAK2-INSR-IRS2 and Akt in the liver, thereby promoting hepatic insulin signaling and alleviating hepatic glucose production and insulin resistance. Additionally, GP treatment ameliorated hepatic inflammation, as evidenced by decreased expression of F4/80, TNF-α, and IL-6. While previous reports attributed the effects of GP on alternative pathways, including the promotion of autophagy via the p62/NF-κB/GLUT4 axis in HepG2 cells [8], enhancing AMPK-mediated Txnip degradation in 3T3-L1 adipocytes [9], regulating RBP4 [10], modulating adipocytokine and PPAR-α activity [7], activating antioxidant enzymes [11], and reducing TNF-α and IL-6 [12], no prior study has focused on insulin signaling as a central mechanism in an obese mouse model. This study includes multiple levels of analysis, including gene expression, serum biomarker analysis, histological evaluation, and assessment of organ weights using an HFD-induced NASH mouse model, providing insights into the therapeutic potential of GP in addressing metabolic disorders.

The significant increases in body, liver, and epididymal fat pad weights in the HFD group were effectively mitigated by GP, suggesting its potential to alleviate visceral obesity and NAFLD. In line with these findings, GP treatment inhibited HFD-induced fat accumulation in both adipose and liver tissues, as confirmed by the quantitative analysis of adipocyte size and hepatic lipid droplets, respectively. Obesity is a major contributor to insulin resistance, primarily through the induction of low-grade chronic inflammation which further aggravates metabolic dysfunction [1]. This inflammatory state involves the infiltration of immune cells into insulin-sensitive organs, such as adipose tissue and the liver, leading to the secretion of pro-inflammatory cytokines that interfere with insulin signaling [13]. Under obese conditions, a shift toward pro-inflammatory M1 macrophage polarization occurs, accompanied by increased production of cytokines such as TNF-α and IL-6, both of which disrupt insulin signaling and contribute to insulin resistance [14,15]. IFN-γ also promotes M1 macrophage polarization and enhances the expression of TNF-α and IL-6, thereby exacerbating systemic inflammation and insulin resistance [16]. MAPK14, also known as p38α, plays a key role in macrophage-mediated inflammation by regulating TNF-α and IL-6 expression and modulating insulin signaling [17]. In contrast, IL-10 is an anti-inflammatory cytokine that protects insulin sensitivity by suppressing pro-inflammatory cytokine production [18]. Our results indicate that GP treatment effectively alleviates inflammation in both adipose tissue and liver, through significantly reducing TNF-α and F4/80 in adipose tissue and TNF-α, F4/80, and IL-6 in the liver. However, IL-10, MAPK14, and IFN-γ expression remained unchanged, suggesting that GP primarily exerts its anti-inflammatory effects by reducing pro-inflammatory cytokine production and macrophage infiltration, rather than by enhancing anti-inflammatory cytokines or modulating MAPK14-related signaling. As a previous in vitro study using LPS-stimulated RAW 264.7 macrophage cells showed that GP suppressed TNF-α and IL-6 expression by inhibiting upstream pathways including the phosphorylation of NF-κB inhibitor α (IκB-α) and nuclear translocation of NF-κB [19], the inhibitory effects of GP on TNF and IL-6 observed in our study may be attributed to suppression of upstream NF-κB pathways. Nevertheless, further studies are required to confirm this mechanism and to determine whether GP directly interacts with TNF or interleukins, or whether such complexes are subsequently degraded.

GP administration significantly reduced fasting glucose levels, improved glucose tolerance as shown by OGTT, and enhanced insulin sensitivity as supported by HOMA-IR, compared to the HFD group. To explore the underlying mechanisms, we assessed the mRNA expression of key insulin signaling genes, including INSR, IRS-1/2, JAK2, Akt1, and IGF1R. The phosphatidylinositol 3-kinase (PI3K)/Akt pathway is pivotal in mediating insulin action via IRS proteins [20]. Impaired insulin signaling—particularly reduced IRS-1/2 tyrosine phosphorylation—is associated with defective glycogen synthesis and increased hepatic glucose output [21]. Normally, insulin and IGF-1 activate their receptors, initiating the IRS–PI3K–Akt cascade, where Akt promotes glucose uptake, suppresses gluconeogenesis, and enhances glycogen synthesis [22]. Thus, upregulating INSR, IRS-1, and IRS-2 improves insulin signaling. Conversely, excessive JAK2 activity may impair this process; hepatocyte-specific JAK2 deletion alleviates HFD-induced steatohepatitis and insulin resistance [23], while its overactivation suppresses Akt signaling [24], potentially exacerbating insulin resistance. Our study revealed that GP treatment significantly upregulated INSR, IRS-2, and Akt1 expression in liver tissue compared to the HFD group while concurrently suppressing JAK2 expression. This dual effect—enhancing IRS signaling while reducing JAK2-mediated inflammation—suggests that GP may effectively activate the INSR–IRS2–Akt signaling axis, ultimately improving insulin sensitivity and mitigating insulin resistance. Further investigation of PI3K expression and activation is warranted to confirm GP’s role in modulating the IRS–PI3K–Akt signaling cascade.

In an insulin-resistant state, impaired suppression of hormone-sensitive lipase triggers excessive lipolysis and elevates levels of FFAs [25,26], which are re-esterified in the liver into TGs, contributing to hepatic steatosis [27]. Increased hepatic TGs promote very-low-density lipoprotein cholesterol (VLDL-C) secretion and reduce HDL-C via hepatic lipase activity, thereby accelerating NAFLD progression to NASH and fibrosis [28]. GP treatment significantly improved lipid profiles (TG, LDL-C, FFA, phospholipids), suggesting an ameliorative effect. PPAR-α, a key lipid metabolism regulator activated by elevated FFAs [29], induces FGF21, which enhances fatty acid oxidation and glucose homeostasis [30]. FGF21 further activates AMPK, and this stimulates PPAR-α, forming a positive feedback loop that supports lipid clearance and energy balance [31]. In our study, GP treatment significantly decreased the expression of PPAR-α and FGF21 compared to the HFD group, suggesting a shift in lipid metabolism regulation.

Histological analysis showed that GP treatment significantly reduced hepatic lipid accumulation and fibrosis areas, suggesting its therapeutic potential for NASH. Along with the histological analysis, GP treatment alleviated elevation in AST and ALT levels, indicating attenuation of liver injury. Peptide-level data supported this by revealing modulation of NASH-related pathways. AMPK, a key player in cellular energy homeostasis, regulates diverse metabolic processes including lipid metabolism, glucose metabolism, inflammation, and oxidative stress [32]. While overnutrition and the consequent obesity-related metabolic diseases induce AMPK downregulation, enhancement of AMPK appears as a highly attractive therapeutic target [33]. To elaborate, hepatic steatosis can be ameliorated by enhancing AMPK by inhibiting de novo lipogenesis and activating fatty acid oxidation, thus improving liver steatosis and insulin resistance. Marked increase in AMPK expression in our study, which is consistent with the previous studies [9,34], may contribute to the inhibition of hepatic steatosis and inflammation, and consequently the alleviation of NAFLD and NASH. Additional molecular mechanisms beyond GP-induced AMPK enhancement require further investigation, including its activators and its downstream enzymes such as acetyl-CoA carboxylase (ACC).

This study has some limitations. Unlike the previous reports [35], GP did not improve TC or HDL-C levels, possibly due to differences in dosage, treatment duration, or the specific mouse model used. Additionally, NRF2 expression was also unchanged, contrasting with earlier findings [34]. This discrepancy may be attributed to different experimental animal models. While the earlier study employed a tyloxapol-induced NAFLD mice model, which induces acute lipid metabolism disorder, our study utilized an HFD-induced NAFLD mice model that reflects the chronic pathophysiology of diet-induced NAFLD. Third, some effects of GP, such as changes in body weight, epididymal fat, liver weight, LDL-C, phospholipid, FFA, AST, ALT levels, hepatic lipid droplet accumulation, fibrosis area, and adipocyte size, were more pronounced in the GP 50 mg/kg group than in the GP 100 mg/kg group, indicating non-dose-dependent effect. Previous studies which investigated improvements in glucose metabolism include a study that utilized a C57BL/6 mice model with high-fat diet and GP injection at 25 or 50 mg/kg [10], a study that demonstrated hepatoprotective effects of GP through oral administration at 25, 50 or 100 mg/kg in the Sprague–Dawley rat model [7], and a study that elaborated ameliorative effects in tyloxapol-induced NAFLD mice model at 50, 75, or 100 mg/kg with a single injection, and they reported dose-dependent responses. While no previous studies have employed the same experimental animal as the present study, our study utilized GP doses of 50 and 100 mg based on the previous studies which utilized mainly rat models [7]. Genipin, a hydrolyzed form of GP by intestinal flora, exhibits both hepatotoxic and hepatoprotective effects [36]. High doses of over 125 mg/kg or prolonged use of genipin may induce liver injury through the production of dialdehyde intermediates and binding to nucleophilic molecules. Thus, the atypical dose–response relationship observed in this study suggests a non-linear relationship between GP dosage and its metabolic effects in this specific model, thereby necessitating further investigation into the pharmacokinetics and pharmacodynamics of GP for the optimal therapeutic window. Fourth, although the pathogenesis of NAFLD and NASH is multifactorial and complex [37,38], the present study focused on specific aspects, highlighting the need for further research to fully elucidate the broader underlying mechanisms. Lastly, while valuable molecular mechanisms were elaborated in this study, further investigations on protein-level validation would provide more comprehensive insights.

**Figure 6:**
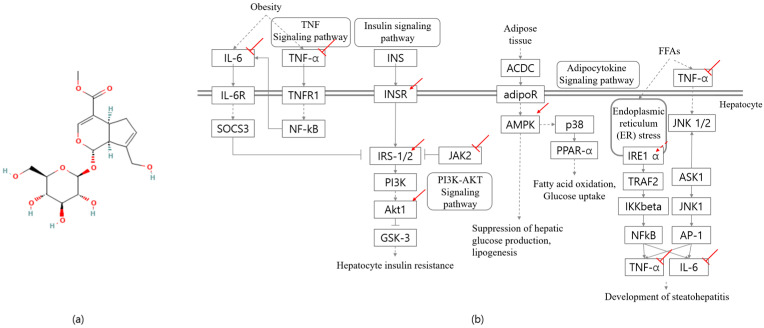
Mechanistic overview of GP’s effects on insulin resistance and hepatic fibrosis. (**a**) Chemical structure of GP. (**b**) Simplified mechanistic pathway illustrating GP’s action against insulin resistance and liver fibrosis.

While previous studies have demonstrated the ameliorative effects of GP on insulin resistance via modulation of NF-κB and GLUT4 in HepG2 cells [8] and regulation of Akt, IRS-1, and GLUT-1 in in 3T3-L1 adipocytes [9], our study extends these findings in several important ways. First, our study was conducted in an in vivo model, enabling a more comprehensive context. Second, we have elaborated more specific upstream pathways underlying the enhancement of AKT, including the INSR–IRS2 and JAK2–IRS2 axes, which have not been fully characterized in the previous studies (Figure 6). Furthermore, our study comprehensively addressed multiple pathological features of NASH beyond impaired insulin signaling, such as inflammation and metabolic dysregulation, offering a broader understanding of GP’s therapeutic potential in liver disease. Given that ectopic lipid accumulation and lipotoxicity-induced inflammation are key drivers of NAFLD and NASH progression [38], GP was shown to alleviate these pathological features by regulating AMPK and inflammatory markers such as F4/80, TNF-α, and IL-6 (Figure 6). Collectively, our findings suggest that GP administration improves insulin signaling, hepatic steatosis, and inflammation, supporting its potential as a therapeutic agent for metabolic liver diseases.

## 4. Materials and Methods

### 4.1. Animals and Drug

Male C57BL/6 mice (6 weeks old, weighing 21–24 g) were purchased from Central Lab Animal Inc. (Seoul, Republic of Korea). The mice were housed under a 12-hour light/dark cycle, with humidity maintained at 40–70%, and provided ad libitum access to food and water. After a one-week acclimatization period, all experimental groups, except for the normal chow (NC) group, had free access to a 60% fat-containing high-fat diet for 17 weeks. GP (CAS RN: 24512-63-8) was purchased from Tokyo Chemical Industry (Tokyo, Japan).

### 4.2. Experimental Group Allocation and Study Design

Male C57BL/6 mice were randomly allocated into groups of five: NC group, control (HFD) group, GP 50 mg/kg (GP50) group, and GP 100 mg/kg (GP100) group. Starting from week 5 of HFD feeding, the HFD group received normal saline, while the GP50 group and GP100 group were orally administered GP at doses of 50 mg/kg and 100 mg/kg, respectively, once daily for 12 weeks. The rationale for the initiation of treatment after 5 weeks of an HFD diet is based on the previous study which reported glucose intolerance present after 1 week of HFD diet and the adipocyte hypertrophy and phenotypic switch of anti-inflammatory M2 to pro-inflammatory M1 macrophages in the adipose tissue after 5 weeks of HFD diet [39]. Thus, initiation of intervention was intended to start at an early yet pathologically relevant stage of insulin resistance. All treatments continued until week 17 of the study.

### 4.3. Weight Measurement of Body, Liver, and Fat Tissue

The body weight of each mouse was measured weekly at a fixed time, starting from the first day of the experiment and continuing for 17 consecutive weeks using an electronic scale (CAS 2.5D, Seoul, Republic of Korea). To ensure accurate measurements and minimize movement, each mouse was placed in a plastic container during weighing. At the end of week 17, the mice were euthanized, and the epididymal fat pad and liver were excised and weighed. The measurements were taken using an electronic balance (CAS 2.5D, Seoul, Republic of Korea).

### 4.4. Assessment on Glucose Tolerance

OGTT was performed in the 17th week of the study. After a 14-hour fasting period (with access to water only), each mouse received an oral dose of glucose at 2 g/kg body weight. Blood samples were obtained from the tail vein at baseline (fasting state) and 30, 60, 90, 120, and 180 min post-glucose administration. Blood glucose levels were measured using a glucose meter Accu-Check Performa with test strips (Roche Korea Company Ltd., Seoul, Korea).

At week 17, insulin levels were obtained using an ultrasensitive mouse insulin ELISA kit (Crystal Chem INC, Elk Grove Village, IL, USA) with collected blood samples. HOMA-IR was utilized for the assessment of insulin sensitivity, using the following equation: HOMA-IR = fasting blood glucose (mg/dL) × fasting insulin (µg/mL) × 0.0717225161669606.

### 4.5. Blood Lipid Profile Test

At week 17, blood samples were collected from the heart to analyze blood lipid profiles. The levels of TC, LDL-C, HDL-C phospholipids, TG, and non-esterified FFAs were measured using an ELISA kit from MyBioSource, San Diego, CA, USA.

### 4.6. Hepatic and Renal Function Tests

To assess liver and kidney function, blood samples were collected from the heart of each mouse during week 17. The samples were then centrifuged at 3000 rpm for 20 min, and the resulting supernatants were stored at −40 °C until analysis. Biochemical analyses for measuring AST, ALT, and serum creatinine levels were performed using an ELISA kit from MyBioSource, San Diego, CA, USA.

### 4.7. Histological Analysis

Tissue samples from the epididymal fat pad and liver were fixed in 10% neutral buffered formalin and then dehydrated sequentially in 70%, 80%, 90%, and 100% ethanol. After fixation, the samples were embedded in paraffin blocks and sectioned into 4 μm thick slices. The sliced tissue sections were placed on gelatin-coated slides, dewaxed in xylene, and then rehydrated using a graded ethanol series (100%, 95%, 80%, and 70%) followed by distilled water for staining. Hematoxylin and eosin (H&E) staining was performed to visualize the tissue morphology. Images of the stained sections were captured using a high-resolution optical microscope (Olympus BX-51, Olympus Optical, Tokyo, Japan) equipped with a camera. The size of adipocytes in the epididymal fat tissue and liver was quantified using ImageJ software (version 1.54k).

To further assess fibrosis in liver tissue, Sirius red staining was performed. Formalin-fixed liver tissues were processed, and 4 µm thick paraffin-embedded sections were stained with 0.1% Sirius Red solution for 60 min. The sections were then rinsed with 100% ethanol, dehydrated, and mounted for analysis. The fibrosis area of the liver tissue was quantitatively analyzed using ImageJ.

### 4.8. RNA Isolation from Tissue

At the end of week 17, following the removal of epididymal fat pad and liver tissue, the samples were wrapped in aluminum foil and stored at −70 °C for subsequent RNA extraction. RNA isolation was carried out using the Mini RNA Isolation II™ Kit (ZYMO RESEARCH, Irvine, CA, USA).

Frozen adipose tissue was thawed and placed in a tube containing 300 μL of ZR RNA buffer aliquot. After homogenization and centrifugation at 100 rpm, 2 mL of the supernatant was transferred to a Zymo-Spin III Column and centrifuged at 2000 rpm for 1 min. Subsequently, 350 μL of RNA wash buffer was added, followed by centrifugation at 2000 rpm for 1 min. The washing process was repeated three times. Finally, 50 μL of RNA-free water was added, and the sample was centrifuged at 1000 rpm to obtain the extracted RNA, which was then stored at −70 °C.

### 4.9. Real-Time Quantitative RT-PCR

Quantitative real-time polymerase chain reaction (qRT-PCR) was performed to examine the gene expression of TNF-α, F4/80, IL-6, IL-10, IFN-γ, FGF21, JAK2, INSR, IRS-1, IRS-2, AKT1, IGF1R, MAPK14, PPAR-α, NRF2, and AMPK in the liver and epididymal fat tissue. Before conducting qRT-PCR, complementary DNA (cDNA) was synthesized using the Advantage RT-for-PCR Kit (Clontech, Mountain View, CA, USA). For cDNA synthesis, 1 μg of RNA extracted from the tissue was mixed with Oligo dT and RNase-free H_2_O, incubated at 70 °C for 2 min, and then allowed to cool. Reverse transcription was initiated by adding a recombinant RNase inhibitor, 10 nM dNTP, MMLV reverse transcriptase, and 5X reaction buffer, followed by incubation at 94 °C for 5 min and 42 °C for 60 min. For PCR amplification, primers, dH_2_O, 2X SYBR reaction buffer, and cDNA were combined and processed using the 7900HT Fast Real-Time PCR System (Applied Biosystems^®^, Waltham, MA, USA). The primer sequences utilized for the experiments are detailed in Table 1.

The threshold cycle (Ct) of each gene was analyzed based on the relative quantification (RQ) method, with EF-1α as the reference gene. The fold-change values were calculated using SDS Software 2.4 (Applied Biosystems^®^, USA) and normalized to the NC group, which was set as 1.

### 4.10. Statistical Analysis

Statistical analyses were conducted using GraphPad PRISM 5 (GraphPad Software Inc., San Diego, CA, USA). Differences among groups were assessed using one-way analysis of variance (ANOVA) followed by Tukey’s post hoc test. Data were expressed as the mean ± standard error (SE). A two-tailed *p*-value < 0.05 was considered statistically significant.

Statistical significance was denoted using number signs (#) for comparisons between the NC group and the HFD group, while asterisks (*) were used to indicate significance between the HFD group and GP groups. The levels of significance were represented as follows: # or * for *p* < 0.05; ## or ** for *p* < 0.01; ### or *** for *p* < 0.001.

## 5. Conclusions

GP reduces body weight and adiposity and ameliorates glucose tolerance, insulin sensitivity, and lipid profiles in HFD-induced obese mice. Hepatic lipid accumulation, hepatic fibrosis, and adipocyte hypertrophy were mitigated in histological analysis. These improvements are associated with the regulation of insulin signaling genes (INSR, JAK2, IRS-2, and AKT1), the metabolic modulator AMPK, and inflammatory markers (F4/80, TNF-α, and IL-6). Through these mechanisms, GP attenuated hepatic fibrosis, potentially delaying NASH progression. Overall, GP alleviates HFD-induced insulin resistance, hepatic steatosis, and inflammation, highlighting its promise as a therapeutic candidate for metabolic liver disorders, including NASH.

## Figures and Tables

**Table 1:** Primer sequences used for RT-PCR.

	Forward Primers	Reverse Primers
F4/80	5′-CTCTGTGGTCCCACCTTCAT-3′	5′-GATGGCCAAGGATCTGAAAA-3′
TNF-α	5′-TTCTGTCTACTGAACTTCGGGGTGATCGGTCC-3′	5′-GTATGAGATAGCAAATCGGCTGACGGTGTGGG-3′
IL-6	5′-AACGATGATGCACTTGCAGA-3′	5′-GAGCATTGGAAATTGGGGTA-3′
IL-10	5′-CGGGAAGACAATAACTGCACCC-3′	5′-CGGTTAGCAGTATGTTGTCCAGC-3′
IFN-γ	5′-ACTGGCAAAAGGATGGIGAC-3′	5′-TGAGCTCATTGAATGCTTGG-3′
MAPK14 p38	GAGCGTTACCAGAACCTGTCTC	AGTAACCGCAGTTCTCTGTAGGT
JAK2	GCTACCAGATGGAAACTGTGCG	GCCTCTGTAATGTTGGTGAGATC
INSR	5′-AGATGAGAGGTGCAGTGTGGCT-3′	5′-GGTTCCTTTGGCTCTTGCCACA-3′
IRS-1	AAGCACCTGGTGGCTCTCTA	TCAGGATAACCTGCCAGACC
IRS-2	ATACCGCCTATGCCTGTCTG	TGGTCTCATGGATGTTCTGC
protein kinase B (Akt)	5′-TGGACTTCCGATCAGGCTCAC-3′	5′-GCCCTTGCCCAGTAGCTTCA-3
IGF1R	5′-CGGGATCTCATCAGCTTCACAG-3′	5′-TCCTTGTTCGGAGGCAGGTCTA-3′
FGF21	5′-ATCAGGGAGGATGGAACAGTGG-3′	5′-AGCTCCATCTGGCTGTTGGCAA-3′
PPAR-α	5′-ACCACTACGGAGTTCACGCATG-3′	5′-GAATCTTGCAGCTCCGATCACAC-3′
NRF2	5′-CTGAACTCCTGGACGGGACTA-3′	5′-CGGTGGGTCTCCGTAAATGG-3′
AMPK	5′-GGTGTACGGAAGGCAAAATGGC-3′	5′-CAGGATTCTTCCTTCGTACACGC-3′

TNF-α, tumor necrosis factor-α; IL-6, Interleukin 6; IL-10, Interleukin 10; IFN-γ, interferon-γ; MAPK14, Mitogen-activated protein kinase 14; JAK2, Janus kinase 2; INSR, insulin receptor; IRS-1, insulin receptor substrate 1; and IRS-2, insulin receptor substrate 2; AKT1, alpha serine/threonine-protein kinase1; IGF1R, insulin like growth factor 1 receptor; FGF21, Fibroblast growth factor 21; PPAR-α, peroxisome proliferator-activated receptor α; NRF2, nuclear factor erythroid 2-related factor 2; and AMPK, adenosine monophosphate-activated protein kinase.

## Data Availability

Data is contained within the article and Supplementary Material.

## Supplementary Materials

The following supporting information can be downloaded at: https://www.mdpi.com/article/10.3390/ijms26168079/s1.
