# Supplementary material for: Geniposide Mitigates Insulin Resistance and Hepatic Fibrosis via Insulin Signaling Pathway
Source: Int J Mol Sci. 2025 Aug 21;26(16):8079. doi: 10.3390/ijms26168079 (PMC12386427; doi:10.3390/ijms26168079)
Supplement: Supplementary file 1 [file ijms-26-08079-s001.zip › ijms-3775096-supplementary.pdf]

Supplementary Materials

Manuscript: Geniposide Mitigates Insulin Resistance and Hepatic Fibrosis via Insulin Signaling Pathway

Authors: Seung-Hyun Oh, Min-Seong Lee, and Byung-Cheol Lee

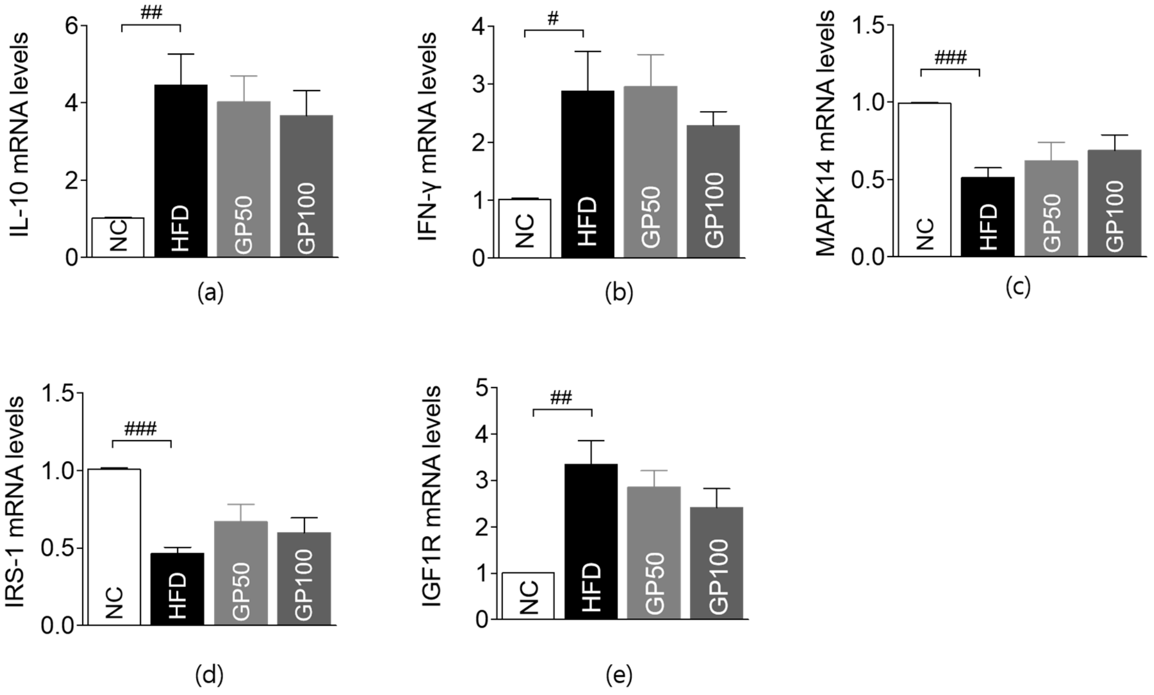

Figure S1. mRNA levels in the liver tissue. (a) IL-10; (b) IFN-γ; (c) MAPK14; (d) IRS-1; (e) IGF1R; Data are described as mean ± SEM; #p < 0.05, ##p < 0.01, ###p < 0.001 compared with NC; NC, normal chow (*n*=5); HFD, high-fat diet (*n*=5); GP50, HFD + geniposide 50 mg/kg/day (*n*=5); GP100, HFD + geniposide 100 mg/kg/day (*n*=5); IL-10, interleukin 10; IFN-γ, interferon-γ; MAPK14, mitogen-activated protein kinase 14; IRS-1, insulin receptor substrate-1; IGF1R, insulin like growth factor 1 receptor.

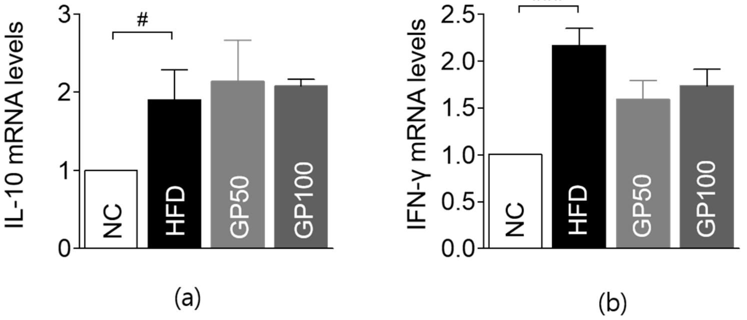

Figure S2. mRNA levels in the epididymal fat tissue. (a) IL-10; (b) IFN-γ; Data are described as mean ± SEM; #p < 0.05, ###p < 0.001 compared with NC; NC, normal chow (*n*=5); HFD, high-fat diet (*n*=5); GP50, HFD + geniposide 50 mg/kg/day (*n*=5); GP100, HFD + geniposide 100 mg/kg/day (*n*=5); IL-10,

Interleukin 10; IFN-γ, interferon-γ.
